# Reply to Magaret and Wald, “Autism Link to Herpes Simplex Virus 2 Antibody in Pregnancy Likely To Be Spurious”

**DOI:** 10.1128/mSphere.00113-17

**Published:** 2017-03-29

**Authors:** Milada Mahic, Siri Mjaaland, Ezra Susser, Michaeline Bresnahan, Xiaoyu Che, Bruce Levin, Mady Hornig, W. Ian Lipkin

**Affiliations:** a Norwegian Institute of Public Health, Nydalen, Oslo, Norway; b Mailman School of Public Health, New York, New York, USA; University of Michigan

**Keywords:** autism, birth cohort, herpes simplex virus, infection, prenatal, serology

## REPLY

We thank Magaret and Wald (1) for comments on our paper that allow us to clarify our experimental design and reemphasize take-home points. They raise four concerns, namely, that (i) there is no correlation between high titers of antibody to HSV and viral replication or HSV acquisition, (ii) the antibody test that we used is not quantitative, (iii) our statistical approach is flawed, and (iv) our conclusions will have an adverse impact on women with a history of HSV infection.

Immune responses to infectious agents are dynamic and decrease with time. With viruses that can reactivate, like HSV-2, reactivation typically results in a rise in antibody titers. We do not have samples acquired prior to midpregnancy, which would be needed to test for a change in antibody titer. However, high antibody titers are consistent with primary infection or reactivation. The bead-based serological platform described in our paper is commonly used to quantitate a wide range of analytes, from cytokines and chemokines to antibodies. A manufacturer may not decide to invest the resources necessary to obtain regulatory approval for a quantitative assay; nonetheless, levels of signal reflect the amount of antibody that binds to beads and can be calibrated.

We used individual statistical tests to address different hypotheses. Only one was ultimately used to test for an association between high HSV-2 antibody levels and ASD risk. All findings, both positive and negative, are reported with exact *P* values, in accordance with American Statistical Association guidance for “full reporting and transparency” (2). We used a logistic-regression model wherein both the linear and the quadratic terms of HSV-2 antibody levels were included as independent variables. The *P* value for the overall adjusted logistic-regression model was 0.0179. The four graphs in Fig. 1 represent the associations afforded by one model at four antibody reference levels, not four different tests. The quadratic term of HSV-2 antibody levels was significant at the 0.03 level, suggesting that HSV-2 antibody levels were associated with ASD risk in a nonlinear format. Due to the presence of the quadratic term, the association between any two levels of HSV-2 antibody varies as a function of the base levels; we simply described these associations at four points.

Many millions of women with a history of HSV-2 infection give birth to children who have normal outcomes. This does not exclude the possibility of a role for infection in the pathogenesis of autism spectrum disorder or other neurodevelopmental disorders. Risk may not be restricted to the central nervous system. Indeed, a recent paper based on a Finnish cohort found a relationship between immunoreactivity to HSV-2 and gastroschisis (3). An adverse outcome probably represents a perfect storm of infection during a period of vulnerability and a robust maternal immune response that results in trafficking of inflammatory molecules across the placenta. The closing paragraph of our paper speculates that risk is not specific to HSV-2 and calls for studies that do or do not replicate our findings and expand to larger serosurveys to test “whether other infectious agents have similar impacts on the incidence of neurodevelopmental disorders.”
